# MEL-18, a tumor suppressor for aggressive breast cancer

**DOI:** 10.18632/oncotarget.4565

**Published:** 2015-06-22

**Authors:** Jeong-Yeon Lee, Gu Kong

**Affiliations:** Department of Pathology, College of Medicine, Hanyang University, Seoul, Republic of Korea

Breast cancer is a heterogeneous disease that is clinically classified into three groups with different therapeutic strategies: estrogen receptor (ER)-positive group receiving endocrine therapy, HER2-amplified group with benefit from HER2-targeted therapy, and triple- negative breast cancer (TNBC; ER-/PR-/HER2-) having only chemotherapy option due to lack of therapeutic targets (reviewed in [1]). Although the mortality of breast cancer has been declining due to the development of targeted therapies, some of breast cancers that harbor aggressive behaviors, such as recurrence, metastasis, and treatment resistance, remain incurable. In particular, cancer stem cells (CSCs), a small subset of cancer cells driving tumor initiation, are considered to display these aggressive phenotypic features. Polycomb group (PcG) protein complexes, which are crucial epigenetic regulators that alter histone modifications, have been associated with breast cancer aggressiveness as key regulators of CSC and tumor metastasis [2]. While the role of BMI-1, a member of PcG proteins also known as a CSC marker, in promoting breast cancer progression has been well established, there is relatively little known about the role of MEL-18, a homolog of BMI-1, in human cancers. As yet, only some studies have implied the opposite role and the distinct expression patterns of BMI-1 and MEL-18 in breast cancer cells and several primary tumors, including breast tumors.

In recent years, accumulating evidence indicates that the loss of the PcG protein MEL-18 is associated with many aggressive behaviors independent of PcG complex functions in breast cancer. MEL-18 has a tumor suppressive function by inhibiting breast tumor growth and angiogenesis as a negative regulator of PI3K-AKT pathway in a BMI-1-independent manner [3]. Moreover, unlike BMI-1, a well-known self-renewal regulator for CSC, MEL-18 loss has promoted breast tumor initiation by inducing breast CSC-like properties via activating the AKT-mediated Wnt/β-catenin and Notch signaling [4]. These results also indicate that AKT has a central role in the regulation of MEL-18 function. Recently, the epithelial–mesenchymal transition (EMT), a key process for tumor invasion and metastasis, has been defined to be a characteristic of stem-like cells [2, 5]. Consistently, our recent study showed that MEL-18 inhibits EMT and tumor invasion via miR-205-dependent downregulation of ZEB1 and ZEB2 expression [6]. Since these EMT markers, as well as Wnt and Notch signaling targeted by MEL-18, have been shown to be associated with both CSCs and EMT regulation, these findings may suggest MEL-18 as a critical molecular linker between EMT and CSC.

The loss of ER function is one of the key signatures for EMT and CSC, as well as aggressive behavior in human breast cancer. Thus in the latest work published in *J Clin Invest*, we have focused more on understanding the mechanisms underlying the role of MEL-18 in ER- mediated hormonal regulation of breast cancer [7]. Among the breast cancer subtypes, TNBC has high similarity to the EMT and CSC phenotypes [1]. Indeed, the loss of MEL-18 expression is associated with TNBC compared with ER or HER2-positive breast cancers, and MEL-18 correlates with the expression of hormonal receptors, ER and PR [7]. Interestingly, MEL-18 loss led to transcriptional downregulation of *ESR1* encoding ER and conferred hormone independence and tamoxifen resistance on ER-positive breast cancer [7]. Together, these results indicate that MEL-18 loss accelerates breast cancer progression from ER-dependent status to more aggressive phenotype with ER loss and independence, supporting the evidence that loss of MEL-18 function is required for acquisition of aggressive phenotype in breast cancer.

Insofar as tamoxifen resistance due to the loss of MEL-18, we suggest two molecular mechanisms, target alteration and bypass of estrogen signaling via ER-independent growth signaling [7]. First, tamoxifen is a selective ER modulator that binds ER to act as a competitive estrogen antagonist. Thus, the lower level of ER expression by MEL-18 loss-mediated transcriptional downregulation of *ESR1* may lead to block the antagonistic effect of tamoxifen on ER in ER-positive breast cancer. It is also notable that MEL-18 regulates *ESR1* expression via modulating the SUMOylation of *ESR1* transcription factors, p53 and SP1, suggesting a strategy for SUMOylation-mediated ER restoration to recover tamoxifen sensitivity. Second, constitutive activation of AKT pathway by MEL-18 loss may overcome the tamoxifen-mediated growth inhibitory effect. Hyperactivation of PI3K/AKT signaling has been implied for hormone-independent breast cancer growth. This may also explain why MEL-18 loss could accelerate ER-positive breast cancer growth despite leading ER loss. Additionally, we assume that expansion of breast CSC accompanied by EMT promotion due to MEL-18 loss may also contribute to tamoxifen resistance in breast cancer.

In summary, we have demonstrated that MEL-18 has a tumor suppressive function in breast cancer via multiple mechanisms, including regulation of signal cascade, miRNA-mediated epigenetic alteration, and SUMOylation-dependent transcriptional regulation in a PcG-independent manner. Loss of MEL-18 might be a critical feature in acquiring aggressive behaviors, including EMT and CSC promotion, hormone independence, and endocrine therapy resistance in breast cancer, suggesting the emerging role of MEL-18 as a new determinant of breast cancer aggressiveness.
